# Gravidity and malaria trends interact to modify *P. falciparum* densities and detectability in pregnancy: a 3-year prospective multi-site observational study

**DOI:** 10.1186/s12916-022-02597-6

**Published:** 2022-11-15

**Authors:** Glória Matambisso, Nanna Brokhattingen, Sónia Maculuve, Pau Cisteró, Henriques Mbeve, Anna Escoda, Judice Miguel, Elena Buetas, Ianthe de Jong, Boaventura Cuna, Cardoso Melembe, Nelo Ndimande, Gemma Porras, Haily Chen, Kevin K. A. Tetteh, Chris Drakeley, Benoit Gamain, Chetan Chitnis, Virander Chauhan, Llorenç Quintó, Beatriz Galatas, Eusébio Macete, Alfredo Mayor

**Affiliations:** 1 Centro de Investigação em Saúde de Manhiça (CISM), Maputo, Mozambique; 2 ISGlobal, Hospital Clínic - Universitat de Barcelona, Barcelona, Spain; 3 Faculty of Infectious and Tropical Diseases, London School of Hygiene & Tropical Medicine, London, UK; 4 Université de Paris, Biologie Intégrée du Globule Rouge, UMR_S1134, Inserm, 75015 Paris, France; 5 Department of Parasites & Insect Vectors, Malaria Parasite Biology and Vaccines, Institut Pasteur, Paris, France; 6 Malaria Group, International Centre for Genetic Engineering and Biotechnology (ICGEB), New Delhi, India; 7 National Directare of Health, Ministry of Health, Maputo, Mozambique; 8 Spanish Consortium for Research in Epidemiology and Public Health (CIBERESP), Madrid, Spain; 9 Department of Physiologic Sciences, Faculty of Medicine, Universidade Eduardo Mondlane, Maputo, Mozambique

**Keywords:** Malaria, Pregnancy, Immunity, Gravidity, Transmission, Parasite densities, Detectability

## Abstract

**Background:**

Low-density *Plasmodium falciparum* infections prevail in low transmission settings, where immunity is expected to be minimal, suggesting an immune-independent effect on parasite densities. We aimed to describe parasite densities in pregnancy, and determine how gravidity and antibody-mediated immunity affect these, during a period of declining malaria transmission in southern Mozambique.

**Methods:**

We documented *P. falciparum* infections at first antenatal care visits (*n* = 6471) between November 2016 and October 2019 in Ilha Josina (high-to-moderate transmission area), Manhiça (low transmission area), and Magude (pre-elimination area). Two-way interactions in mixed-effects regression models were used to assess gravidity-dependent differences in quantitative PCR-determined *P. falciparum* positivity rates (*Pf*PR_qPCR_) and densities, in the relative proportion of detectable infections (pDi) with current diagnostic tests (≥ 100 parasites/μL) and in antimalarial antibodies.

**Results:**

*Pf*PR_qPCR_ declined from 28 to 13% in Ilha Josina and from 5–7 to 2% in Magude and Manhiça. In primigravidae, pDi was highest in Ilha Josina at the first study year (*p* = 0.048), which declined with falling *Pf*PR_qPCR_ (relative change/year: 0.41, 95% CI [0.08; 0.73], *p* = 0.029), with no differences in antibody levels. Higher parasite densities in primigravidae from Ilha Josina during the first year were accompanied by a larger reduction of maternal hemoglobin levels (− 1.60, 95% CI [− 2.49; − 0.72; *p* < 0.001), than in Magude (− 0.76, 95% CI [− 1.51; − 0.01]; *p* = 0.047) and Manhiça (− 0.44, 95% CI [− 0.99; 0.10; *p* = 0.112). In contrast, multigravidae during the transmission peak in Ilha Josina carried the lowest pDi (*p* = 0.049). As *Pf*PR_qPCR_ declined, geometric mean of parasite densities increased (4.63, 95% CI [1.28; 16.82], *p* = 0.020), and antibody levels declined among secundigravidae from Ilha Josina.

**Conclusions:**

The proportion of detectable and clinically relevant infections is the highest in primigravid women from high-to-moderate transmission settings and decreases with declining malaria. In contrast, the falling malaria trends are accompanied by increased parasite densities and reduced humoral immunity among secundigravidae. Factors other than acquired immunity thus emerge as potentially important for producing less detectable infections among primigravidae during marked declines in malaria transmission.

**Supplementary Information:**

The online version contains supplementary material available at 10.1186/s12916-022-02597-6.

## Background

The density of malaria parasites in an individual has implications for the ability to detect the infection, since standard diagnostic test such as microscopy and rapid diagnostic test (RDT) can miss low density infections [1]. Parasite density is considered to be mainly shaped by host factors, in particular immunity [2]. Naturally acquired immunity against *P. falciparum* develops after repeated exposure, with a faster acquisition in high transmission areas [2]. However, most infections in low transmission settings, where immunity is expected to be minimal, are characterized by their low parasite densities [1]. This observation challenges the idea that host immunity is the only determinant of epidemiological malaria features.

Parasite factors such as the number of co-infecting parasite variants that compete within the human host, intrinsic growth rate, virulence, sexual commitment, and fitness may also play a role in shaping parasite densities under different intensities of transmission [3–5]. *Plasmodium* parasites have developed sophisticated mechanisms to adjust to host conditions [6] and seasonal fluctuations [5]. However, most of the evidence supporting this adaptability has been obtained from in vitro and animal models, leaving the role of parasite factors in shaping human infections and their potential to undermine the efficacy of malaria control programs to be determined [4]. In addition, most of longitudinal data on malaria in Africa is based on symptomatic cases [7] and lacks precise estimations of parasite densities, which makes it difficult to study the epidemiological signatures of these parasite adaptations.

Pregnant women attending antenatal care (ANC) clinics constitute an attractive group for monitoring changes in malaria prevalence in the population [8]. A few studies have explored parasite densities in this group, in particular in low transmission settings or during marked declines in transmission, but none have included direct measures of immunity. Infections acquired before pregnancy are maintained at low density by antimalarial immunity developed after life-long encounters with *P. falciparum* [9, 10]. These infections, together with those acquired in pregnancy, multiply to high densities in primigravidae from the end of the 12th week of gestation [9, 11] when the placenta selects *P. falciparum* parasites expressing VAR2CSA that binds to chondroitin sulfate A [12]. Antibodies against VAR2CSA are acquired in a gravidity-dependent manner, and appear to protect multigravid women against severe malaria [9–11]. This pregnancy specific immune response reflects exposure in very specific time windows (i.e., the pregnancy period) and might be utilized to gain a better understanding of the role of immunity in controlling parasite densities.

There is little knowledge on the relationship between intensity of malaria transmission, parasite densities, and immunity, especially among pregnant women and in contexts of declining transmission. Here, we aimed to describe parasite densities in pregnancy, and how gravidity and humoral immunity affect these, during a period of declining malaria in southern Mozambique. To address this, we undertook a 3-year prospective observational study among pregnant Mozambican women at first ANC visits in southern Mozambique. We applied molecular methods to quantify *P. falciparum* infections and antibody levels in women from a historically moderate transmission setting (Ilha Josina) [13], a setting that rapidly transitioned from moderate to low malaria transmission after an elimination initiative (Magude) [14], and a historically low malaria transmission setting (Manhiça). Using an interaction analysis, we tested the hypothesis that pregnancy-specific immunity is the major factor controlling parasite densities in malaria-exposed multigravidae, whereas immune-independent parasitological factors dominate parasite densities in anti-VAR2CSA-naïve primigravidae.

## Methods

### Study area and population

This 3-year prospective observational study was conducted between November 2016 and November 2019 at ANC clinics in Manhiça Sede District Hospital, Ilha Josina Health Center (Manhiça District), and Magude Sede Health Center (Magude District), in Maputo Province, southern Mozambique (Additional File 1: Fig. S1). The region is characterized by its subtropical climate with a hot and rainy season from November to April and a cool and dry season during the rest of the year. Overall, malaria transmission is low in Manhiça District, with rapid diagnostic test (RDT)-based parasite rates (PR_RDT_) in the community of 6.1% and 1.4% in 2017 [15] and 2018 (B. Galatas, personal communication), respectively. PR_RDT_ in Ilha Josina, a river island in the confluences of the Incomati River with historically high-to-moderate transmission levels [13], was 18.1% in 2017 and 3.7% in 2018 (B. Galatas, personal communication). Magude District, located 102 km from Manhiça, is a low transmission area (PR_RDT_: 2.6% in 2017 [15] and 1.4% in 2018 [14]) resulted from the deployment of an intensive package of malaria elimination interventions since 2015 [14]. Indoor residual spraying was deployed annually in the study areas.

### Recruitment and data collection

Pregnant women at their first routine ANC visit were invited to participate in the study if resident in the study area. A fingerprick blood drop was collected onto filter papers (dried blood spots [DBS]) with the participant’s consent. A brief form, including visit date, age, gravidity, gestational age based on the fundal height measurement, area of residence, and recent movements was completed. HIV status was recorded from the maternal health card. In case of an unavailable record, an HIV serological rapid test was done according to standard procedures for voluntary counseling and testing. Maternal hemoglobin levels were determined by HemoCue® Hb 201 + (HemoCue AB, Angelholm, Sweden).

### Parasitological determinations

DNA extracted from DBS was used for detection and quantification of *P. falciparum* in duplicate using real-time quantitative PCR (qPCR) assay targeting the 18S ribosomal RNA gene [16]. Experiments were repeated if the qPCR efficiency or the positive control were out of the range obtained by the average ± 3 standard deviations (SD; Additional File 1: Fig. S2). Positive DBSs were used for Illumina next generation sequencing of PCR amplicons targeting 101 single nucleotide polymorphisms (SNPs) in the parasite genome [17]. The 101-SNP barcode was used to estimate the number of distinct genotypes within the infection (complexity of infection [COI]) using the program COIL [18].

### Immunoglobulins G determinations

Immunoglobulin G (IgG) eluted from 5760 available DBS were quantified using the xMAP technology and the Luminex 100/200 System (https://www.luminexcorp.com) [19]. The multiplex suspension array panel included VAR2CSA antigens (Duffy binding-like recombinant domains DBL3-4 [20] as well as peptides targeting the NTS region [P1] and ID1 [P8 and PD] [19]), non-pregnancy specific antigens (19-kDa fragment of the merozoite surface protein-1 [MSP1_19_] [21], region II/F2 of erythrocyte-binding antigen-175 [EBA175] [22], full-length *P. falciparum* reticulocyte-binding homologue protein 2 and 5 (PfRH2 [23] and PfRH5 [24])), and biomarkers of recent *P. falciparum* exposure [25] (gametocyte exported protein 18 [GEXP18], acyl CoA synthetase 5 [ACS5] ag3, early transcribed membrane protein 5 [ETRAMP5] ag1 and heat shock protein 40 [HSP40] ag1). Information about antigens, procedures for reconstitution of DBS and quality control, bead-based immunoassay, and data normalization are described in Additional File 1: Fig. S2 and Additional File 2: Supplementary Methods [26, 27].

### Sample size calculation and data analysis

We estimated that the molecular detection of *P. falciparum* in all DBS collected from Ilha Josina (*n* = 250/year) and a random selection of approximately 2700 samples per year in both Magude and Manhiça would allow for the estimation of the 95% confidence interval (95%CI) of annual *P. falciparum* positivity rates between 20 and 5% in each of the 3 sites, with a margin of error lower than or equal to the expected positivity rate. Age was categorized as either younger than 18 years or 18 years and older, season as rainy (November to April) or dry (May to October) and year of recruitment from November to October in 2016–2017 as year 1, 2017–2018 as year 2,or 2018–2019 as year 3. Women reporting to have changed their residence area during their pregnancy, as well as women with missing information on gravidity, residence area or HIV, were dropped from the analysis. Detectable infections (Di) were defined as those with parasite densities equal or above 100 parasites/μL, which is the detection limit commonly used for standard RDTs [28], and expressed relative to the total of infections detected by qPCR (proportion of Di [pDi]).

Categorical and continuous data were compared between sites, gravidity groups, and time periods using Fisher’s exact test and Student *t*-test, respectively. Multilevel mixed-effects regression models with random intercept at site level were estimated to assess associations with qPCR positivity and pDi (logistic) and log-transformed parasite densities and mean fluorescence intensities (MFIs) as well as hemoglobin levels (linear). All models were estimated using transmission level (site) and gravidity (first pregnancy and two or more previous pregnancies) as independent variables and were adjusted for season, HIV status, residence in village or rural area, and place where molecular analysis was conducted (Mozambique or Spain). The hypothesis that the control mechanisms of *P. falciparum* infection in pregnancy are gravidity-dependent was assessed including an interaction term between gravidity and transmission levels (by site and period) in the models. Secondary analyses, which included genetic complexity of infections and secundigravidae, were assessed using linear and mixed-effects regression models, respectively. The significance level was set at 0.05. All analyses were performed using the Stata statistical program version 16 (Stata Corporation, College Station, TX, USA).

## Results

### Characteristics of study participants

Among the 10,439 first ANC visits recorded in the three antenatal clinics from November 2016 to November 2019, 8745 (8464 women) were included in the study. Not residing in the study area was the main reason for exclusion (Additional File 1: Fig. S3). No major differences were observed between the women included in the study and the 6471 selected for the molecular analyses nor between the women from the 3 study centers (Additional File 3: Table S1). Overall, 8% (518/6471) of the pregnant women presented at the ANC clinic during their first trimester (mean gestational age: 20.4 weeks; SD: 5.5) and 29% (1872/6471) were HIV-infected. The proportion of women at their first, second, and third or more pregnancies was 27% (1754/6471), 26% (1675/6471), and 47% (3042/6471), respectively.

### *P. falciparum *qPCR positivity rates

**Fig. 1:**
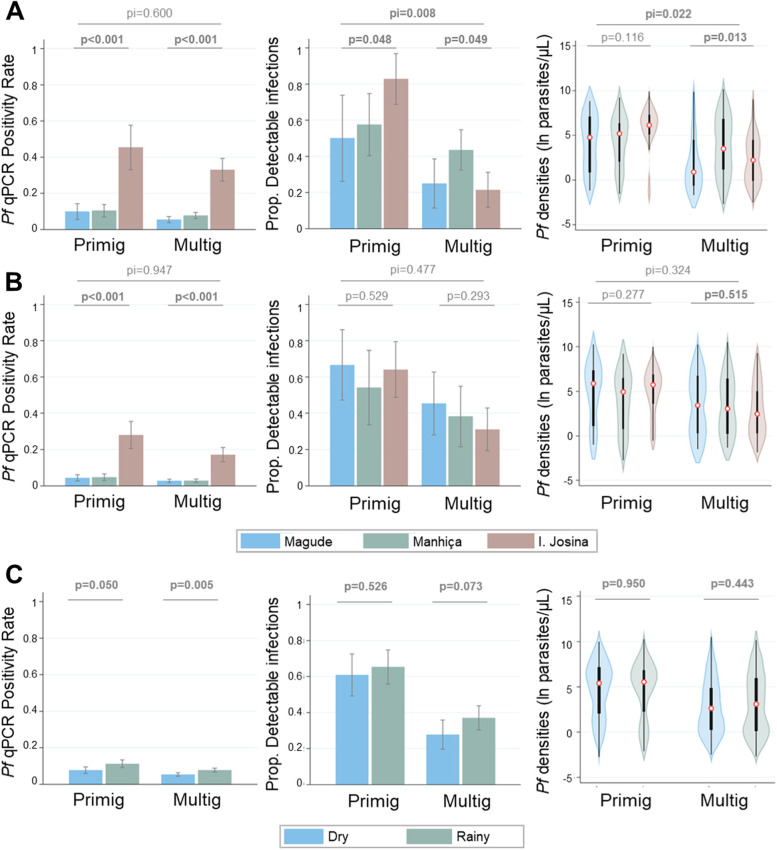
*P. falciparum* positive rates, densities, and proportion of detectable infections between centers by gravidity, study period, and season. Multilevel mixed-effects regression models with a random intercept at site level were estimated to assess associations with detectability (logistic) and log-transformed parasite densities. All models were estimated using transmission level (site) by study period (**A** year 1 and **B** year 2 to 3) or season (**C**) as independent variables and were adjusted for HIV status, residence in village, or rural area and place where molecular analysis was conducted. The model of transmission level (**A** and **B**) included an interaction term to assess the modifying effects of gravidity (first pregnancy [primigravidae: primig] and two or more previous pregnancies [multigravidae: multig]) on the relationship between parasitological outcomes and center of recruitment, with the testing for significance using a Wald test (pI). Parasite densities are represented as violin plots, which include a marker for the median (red circle), a black box indicating the interquartile range and spikes extending to the upper- and lower-adjacent values; the shape represents kernel density estimates

**Fig. 2:**
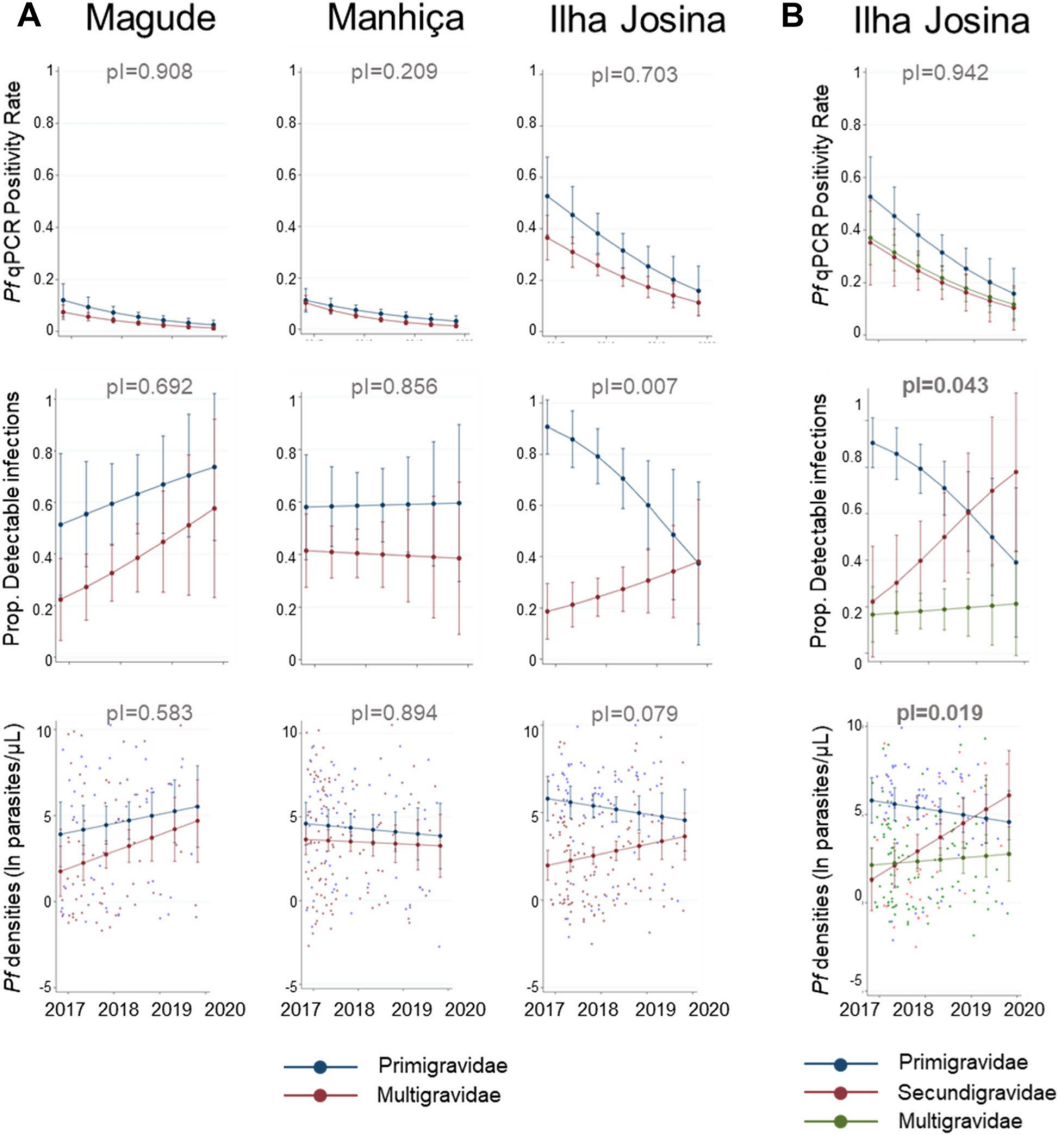
*P. falciparum* positivity rates, parasite density and detectability by gravidity for each health clinic. Estimated *Pf* (*P. falciparum*) qPCR positivity rate and Prop (Proportion) of detectable infections for Magude, Manhiça, and Ilha Josina in primigravidae and multigravidae (**A**). **B** presents the same analysis but including primigravidae, secundigravidae and multigravidae in Ilha Josina. Estimates were obtained from multivariate regression models predicting parasitological outcome in each of the three sites based on visit date, gravidity, and their two-way interactions, adjusted by season, HIV status, residence in a village, or rural area and place where molecular analysis was conducted. The statistical significance of the interaction term (*p* for the interaction [pI]) was assessed using a Wald test and estimates obtained from the coefficients plus the interaction and the standard error by the delta method

A total of 483 (7.5%) out of the 6471 blood samples were positive for *P. falciparum* by qPCR (26% [199/770] in Ilha Josina, 6% [169/3044] in Manhiça, and 4% [115/2657] in Magude, *p* < 0.001; Table 1). Fifty-five percent of the infections (268/483) were detected in the first year (November 2016 to October 2017). qPCR-confirmed *P. falciparum* infections were more likely in first pregnancies (adjusted odds ratio [aOR] 1.65, 95% CI [1.30; 2.11], *p* < 0.001; Fig. 1), during the rainy season (aOR 1.44, 95% CI [1.17; 1.78], *p* < 0.001; Fig. 1), and in women residing in rural settings (aOR 1.99, 95% CI [1.37; 2.87], *p* < 0.001). On average, PfPR_qPCR_ declined annually by 42% (95% CI [30–55], *p* < 0.001) in Ilha Josina (from 28% in year 1 to 13% in year 3), 44% (95% CI [30–57], *p* < 0.001) in Magude (from 5 to 2%), and 45% (95% CI [33–56], *p* < 0.001) in Manhiça (from 7 to 2%; Additional File 3: Table S2). These declines were similar between primigravid and multigravid women (*p* for the interaction [pI] > 0.05; Fig. 2 and Additional File 3: Table S3). The genetic complexity of infections among primigravid women during the first year was lower in Manhiça (1.17, SD 0.39; *n* = 12) compared to Ilha Josina (1.57, SD 0.59; *n* = 23) and Magude (1.60, SD 0.55; *n* = 5; *p* = 0.039), while no differences were observed among multigravidae nor during second and third years (Additional File 1: Fig. S4).

**Table 1:** *P. falciparum* positivity rates, parasite densities, and detectable infections at first antenatal care visit by studied factors

	**PfPR**_**qPCR**_		***Pf* density**		**pDi**	
	***n*** = **6471**		***n*** = **483**		***n*** = **483**	
	***n*/*N* (%)**	***p***	**GM (SD)**	***p***	***n*/*N* (%)**	***p***
**Clinic**						
**Magude**^**a**^	115/2657 (4)	** < 0.001**	30.9 (109.9)	0.482	50/115 (43)	0.416
**Manhiça**^**b**^	169/3044 (6)		51.5 (34.1)		79/169 (47)	
**Ilha Josina**^**c**^	199/770 (26)		34.1 (113.5)		83/199 (42)	
**Period**						
**November 16–October 17**	268/2511 (11)	** < 0.001**	31.5 (102.5)	0.309	111/268 (41)	0.236
**November 17-October 18**	137/2031 (7)		51.5 (162.7)		67/137 (49)	
**November 18-October 19**	78/1929 (4)		34.1 (113.5)		34/78 (44)	
**Village**						
**Yes**	447/6018 (7)	** < 0.001**	36.8 (120.2)	0.989	16/36 (44)	0.898
**No**	36/453 (8)		34.9 (104.4)		196/447 (44)	
**Season**						
**Rainy**	295/3390 (9)	**0.001**	38.9 (128.7)	0.054	75/188 (40)	0.448
**Dry**	188/3081 (6)		33.3 (104.9)		137/295 (46)	
**Age (years)**						
** < 18**	68/670 (10)	0.776	104.8 (313.7)	0.124	45/68 (66)	0.377
** ≥ 18**	415/5801 (7)		30.9 (100.4)		167/415 (40)	
**Gravidity**						
**Primigravid**	167/1754 (10)	** < 0.001**	103.3 (316.9)	** < 0.001**	106/167 (63)	** < 0.001**
**Multigravid**^d^	316/4717 (7)		21.2 (67.9)		106/316 (34)	
**Trimester of gestation**						
**1st**	35/518 (7)	0.694	72.7 (240.6)	0.371	18/35 (51)	0.236
** > 1st**	448/5953 (8)		34.8 (112.4)		194/448 (43)	
**HIV**						
**Infected**	114/1872 (6)	0.078	65.4 (207.6)	**0.004**	56/114 (49)	**0.001**
**Uninfected**	369/4599 (8)		30.7 (99.5)		156/369 (42)	

*N* indicates the number of all samples analyzed by qPCR and *n* the number of qPCR-positive infections. Detectable infections (≥100 parasites/µL) are expressed relative to the total of infections detected by qPCR (N). The *p*-value indicates the statistical significance obtained from the multivariate model adjusted by all the variables included in the table

*GM* Geometric mean, *pDi* Proportion of detectable infections, *SD* Standard deviation

^a^*N* = 907 (year 1), 858 (year 2) and 892 (year 3)

^b^*N* = 1328 (year 1), 893 (year 2) and 823 (year 3)

^c^*N* = 276 (year 1), 280 (year 2) and 214 (year 3)

^d^121 secundigravidae (38.1 [SD 132.1] parasites/µl; 56/121 [46.3%] detectable infections) and 195 with 3 or more previous pregnancies (14.8 [SD 43.9] parasites/µl with 50/195 [25.6%] detectable infections)

### *P. falciparum* densities and detectability

Geometric mean of parasite density was 36.7 parasites/μL (SD 118.9). Forty-four percent (212/483) of the qPCR-detected infections were equal or above 100 parasites/μL and thus considered as infections detectable by conventional RDTs. Parasite densities (*p* = 0.004) and the proportion of detectable infections (*p* = 0.001) were higher among HIV-infected than HIV-uninfected women. Densities were 5.4-fold (95%CI [2.7; 10.6], *p* < 0.001) higher in primigravidae than multigravidae (Table 1); this increase in density was larger in Ilha Josina (16.7, 95% CI [6.3, 33.3], *p* < 0.001) than in Magude (6.3, 95% CI [1.9, 20.0], *p* = 0.002) and Manhiça (1.9, 95% CI [0.7, 5.3], *p* = 0.195; Additional File 3: Table S2). Similar trends were observed in the pDi (Table 1 and Additional File 3: Table S2).

At the peak of *P. falciparum* positivity rates (November 2016 to October 2017), pDi was the highest among primigravidae in Ilha Josina (83%) compared to those in Magude (50%) and Manhiça (58%; *p* = 0.048; Fig. 1 and Additional File 3: Table S4). In contrast, pDi among multigravidae in the first year was the highest in Manhiça (44%) compared to Ilha Josina (21%) and Magude (25%, *p* = 0.049; pI = 0.008; Fig. 1 and Additional File 3: Table S4). Thereafter, pDi in Ilha Josina decreased among primigravidae (average proportional annual change of 0.41, 95% CI [0.08; 0.73]; *p* = 0.029) and tended to increase among multigravidae (1.47, 95% CI [0.68; 2.25]; *p* = 0.161; pI = 0.007; Fig. 2A and Additional File 3: Table S3), mainly driven by the increase among secundigravidae (2.43, 95% CI [0.87–6.76], *p* = 0.089; Fig. 2B and Additional File 3: Table S5). The pDi in Manhiça and Magude remained stable (0.94, 95% CI [0.63; 1.44]; *p* = 0.784) or tended to increase (1.57, 95% CI [0.95; 2.62]; *p* = 0.081), respectively, with no evidence of a modification by gravidity (Fig. 2A and Additional File 3: Table S4). Parasite densities followed the same trends as pDi (Figs. 1, 2 and Additional File 4: Supplementary Results).

### Immunoglobulin G levels against* P. falciparum* antigens

**Fig. 3:**
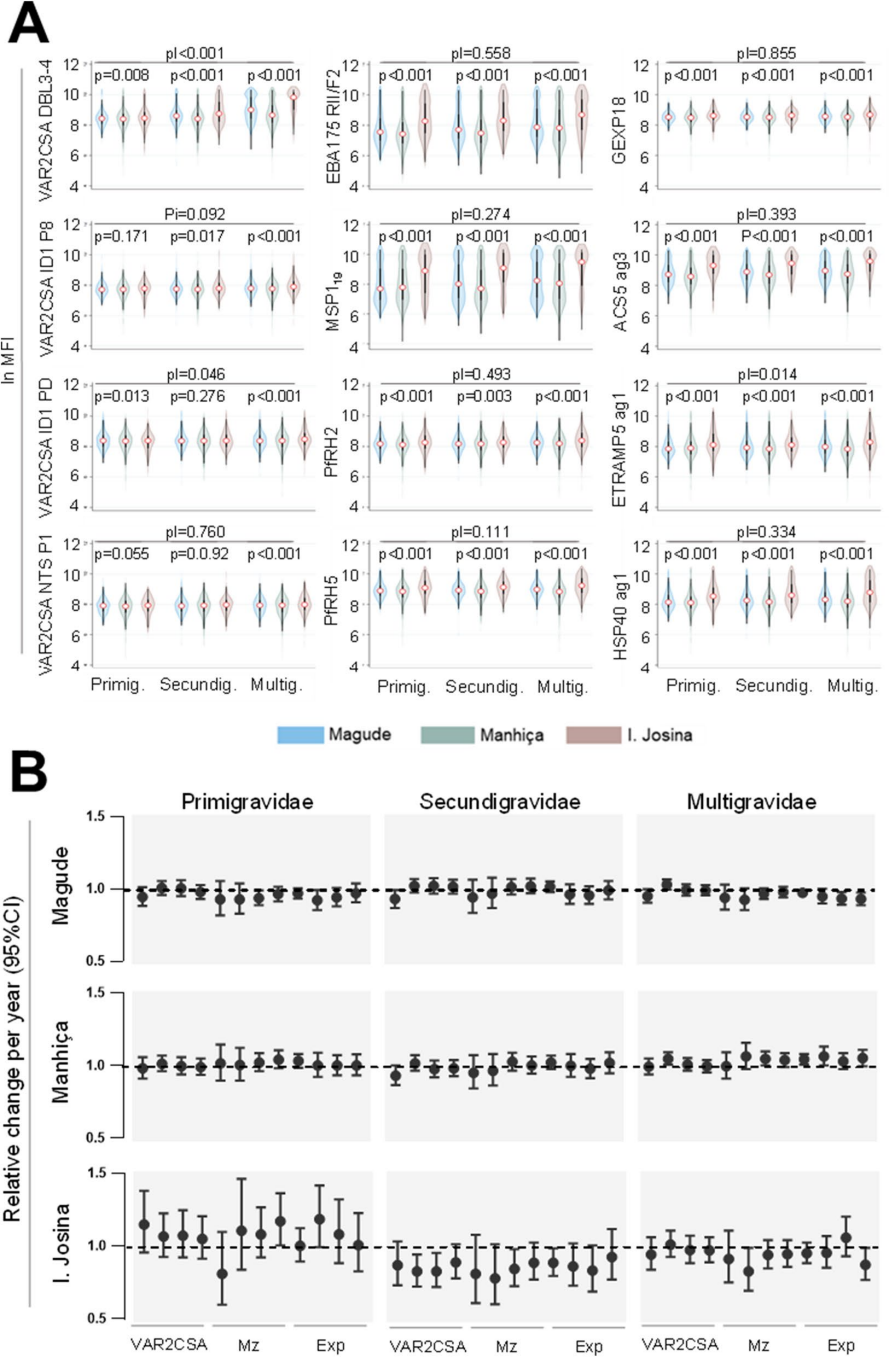
Immunoglobulin G levels against *P. falciparum* antigens. **A** Violin plots of log-transformed antibody levels (MFI) between centers and by gravidity (Primig: primigravidae, Secundig: secundigravidae; Multig: multigravidae). **B** The relative change per year (dot) and 95%CI (T bar) of antibody levels (VAR2CSA, Merozoite [Mz] and recent exposure markers [Exp]), by center and gravidity. Relative change and *p* values were obtained from the multivariate regression models adjusted by season, HIV status and residence in a village or rural area, which included an interaction term to assess the modifying effects of gravidity, with the testing for significance using a Wald test (pI). Dashed lines in **B** represents the relative change per year of 1 (no change)

Antibody levels were higher among women from Ilha Josina (all antigens), HIV-infected (all antigens except ACS5), those residing in rural settings (DBL3-4, RII/F2-EBA175, PfRH2, PfRH5, GEXP18, ACS5, ETRAMP, HSP40), and multigravidae (DBL3-4, P1, MSP1_19_, RII/F2-EBA175, GEXP18 and ACS5; Fig. 3 and Additional File 1: Fig. S5). Antibody levels against DBL3-4 (*p* = 0.013), P8 (*p* = 0.003), P1 (*p* = 0.029), and GEXP18 (*p* = 0.048) declined during the 3-year study period. Since *P. falciparum* infection was associated with increased antibody levels against all the antigens (Additional File 1: Fig. S5), women with *P. falciparum* infection were excluded from further analysis to discard antibody boosting by active infections. Antibodies against DBL3-4 were similarly low in primigravidae women from three sites but increased in secundigravidae and multigravidae from Ilha Josina compared to Manhiça and Magude (pI < 0.001; Fig. 3A). In contrast, antibodies against non-pregnancy-specific antigens were similarly high in all women from Ilha Josina regardless of gravidity. Antibody levels remained constant throughout the 3-year study period in women from Magude and Manhiça, while a reduction in antibody levels was observed among secundigravidae from Ilha Josina (annual relative change of 0.83, 95% CI [0.72; 0.94], *p* = 0.004 for P8-VAR2CSA; 0.83, 95% CI [0.72; 0.96], *p* = 0.009 for Pd-VAR2CSA; 0.84, 95% CI [0.73; 0.98], *p* = 0.026 for PfRH2, and 0.89, 95% CI [0.0.79; 0.99], *p* = 0.026 for GEXP18; Fig. 3B and Additional File 3: Table S6).

### Clinical impact of *P. falciparum* infections

**Fig. 4:**
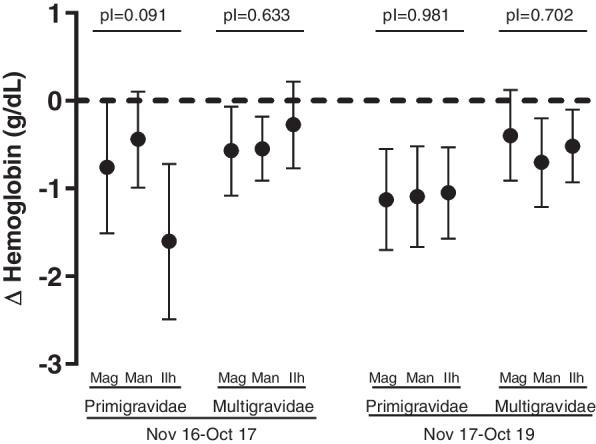
Impact of *P. falciparum* infections on maternal hemoglobin levels. *p* values were obtained from multivariate linear models adjusted by gravidity, season, HIV status, residence in a village or rural area, and place where molecular analysis was conducted. The modification of the associations by gravidity was assessed by including interaction terms into the regression models, testing for significance using a Wald test (pI) and combining the coefficients plus the interaction and the standard error by the delta method. Mag, Magude; Man, Manhica; Ilh, Ilha Josina

*P. falciparum* infection was associated with a reduction of 0.65 g/dL of hemoglobin (95% CI [− 0.80; − 0.50], *p* < 0.001), which was larger among primigravidae (− 0.93 g/dL, 95% CI [− 1.19; − 0.69], *p* < 0.001) than multigravidae (− 0.49 g/dL, 95% CI [− 0.68; − 0.32], *p* < 0.001; pI = 0.004). Among primigravidae, the largest reduction was observed in women from Ilha Josina (− 1.60, 95% CI [− 2.49; − 0.72; *p* < 0.001), then Magude (− 0.76, 95% CI [− 1.51; − 0.01]; *p* = 0.047) and Manhiça (− 0.44, 95% CI [− 0.99; 0.10; *p* = 0.112; pI = 0.091; Fig. 4).

## Discussion

This is the first study to provide epidemiological and immunological evidence of non-immune factors that influence parasite densities when malaria burden falls from high-to-moderate to low levels. Between November 2016 and November 2019, *P. falciparum* qPCR-positivity rates at first ANC visit in southern Mozambique declined from 28% in Ilha Josina, 7% in Manhiça, and 5% in Magude to 13%, 2%, and 2%, respectively. Decreasing transmission in these three different epidemiological settings were followed by changes in parasite densities which are gravidity-dependent. Among secundigravidae in Ilha Josina, parasite densities and the relative abundance of detectable infections increased with declining malaria, as expected from les malaria exposure in the previous pregnancies. However, falling parasite rates were associated with declining parasite densities among anti-VAR2CSA-naïve primigravidae, without major changes in pregnancy-specific and general immunity. Therefore, factors other than acquired immunity produce less detectable, lower-density infections among pregnant women over short but marked declines in malaria burden.

Trends of parasite densities among multigravid women are consistent with the role of pregnancy-specific immunity in controlling parasite densities [16, 29]. The proportion of detectable infections was lower among multigravid women from areas with recent high-to-moderate transmission (Ilha Josina and Magude), compared to primigravidae in respective regions. As *P. falciparum* rates fall in Ilha Josina, parasite densities and detectability in secundigravidae increased to levels observed in primigravidae. These changes in a short period of 3 years are accompanied by reductions in antimalarial antibodies, both pregnancy-specific and generally, in secundigravidae [16], presumably reflecting a reduction in parasite exposure in the previous first pregnancy and a waning of acquired immunity [30]. In contrast, no gravidity effect or change during the study period was observed in parasite densities and pregnancy-specific immunity among women from the historically low transmission setting in Manhiça.

Parasitemia in first pregnancy follows the opposite patterns of multigravid women, pointing to differences in the factors that determine parasite densities among women without pregnancy-specific immunity. When *P. falciparum* rates were at their highest levels (first year of the study), parasite densities and detectability, as well as the infection-associated reduction in hemoglobin, were higher among primigravidae from Ilha Josina, than those from Magude and Manhiça. Thereafter, parasite densities among primigravidae from Ilha Josina decreased as parasite rates dropped. This shift in parasite densities was not accompanied by changes in VAR2CSA-specific antibodies, which were similarly low among primigravid women in the three settings [31], as expected from the limited VAR2CSA exposure at the pregnancy onset (i.e., before the placenta develops [9, 11]). It can also not be explained by general anti-malarial immunity acquired before of pregnancy, as levels of antibodies against non-pregnancy-specific antigens were correlated with parasite rates, which was expected to affect densities reversely. More recent infections in Ilha Josina [32] are also unlikely to explain the higher observed parasite densities, as this effect is expected to manifest independently of gravidity and in the rainy season, when the force of infection is larger, compared to the dry season. Instead, the data suggests the role of factors other than acquired immunity in regulating parasite densities in primigravid women from Ilha Josina during marked epidemiological transitions. The higher number of genetically different parasites among infected primigravidae from Ilha Josina compared to those from Manhiça is in line with the increase in parasite clones and multiplication rates as transmission increases suggested from previous studies [3, 33]. The immunity developed during previous pregnancies may mask these non-immune factors in multigravid women. Further studies are needed to identify the molecular mechanisms of the immune-independent control of parasite densities and their potential impact on balancing parasite replication and gametocyte production, as suggested by evolutionary and experimental models [3, 4].

The results of this study have several public health implications. First, the proportion of *P. falciparum* infections that can be detected with currently available diagnostic tools varies substantially depending on the intensity of transmission and gravidity. The proportion of detectable infections in primigravidae increased from 50 to 83% in areas with a PfPRq_PCR_ of 4% and 28%, respectively. In contrast, detectable infections in multigravidae decreased from 44 to 21% with increasing parasite rates. Therefore, levels and trends of malaria burden, as well as fertility rates, are expected to affect the sensitivity to detect *P. falciparum* infections and as a consequence the efficiency of screen-based strategies [34], as well as the contribution of pregnant women in sustaining transmission [35]. Second, the adverse effects of *P. falciparum* infections over periods in which malaria prevalence declines may increase among multigravid women as immunity wanes and parasite densities increase, but decrease among primigravidae as non-immune factors reduce parasite densities. Third, the results of this study highlight the value of monitoring malaria in pregnancy as an independent measure of malaria burden [8] that can capture geographical and temporal malaria trends. Fourth, the identification of factors other than acquired immunity which determine densities as transmission changes [5] may allow the improvement of surveillance approaches and the development of new antimalarial interventions. Finally, incorporating non-immune factors in mathematical models of malaria in pregnancy [9, 34, 35] may improve their efficacy in describing the host-parasite biology and malaria transmission dynamics.

This analysis has several limitations. First, inaccessibility of the placenta at booking visits prevents the direct study of parasite dynamics in that organ; however, parasite densities in peripheral and placental blood have been shown to follow similar trends [16, 29]. Second, the low number of *P. falciparum* infections in the low transmission settings of Magude and Manhiça limited the ability to stratify women with two or more pregnancies in the analysis. Third, genotyping of parasite infections was not successful for low-density infections, which represents a large proportion of available samples. Fourth, age-dependent effects might have been attributed to pregnancy-specific immunity, given the correlation between age and gravidity. However, previous modeling data [34] suggests that pregnancy, rather than age, is the major determinant of the observed patterns. Fifth, it is not possible to generalize the observations of this study to non-pregnant individuals, although similar reductions in parasite densities with declining transmission have been observed in the general population [36, 37]. Finally, the study did not assess genetic, behavioral, socio-economic, and environmental factors which may contribute to the different malaria burden [32]. However, these are not expected to vary substantially in the study populations, since they were relatively closely located in the south of Mozambique.

## Conclusions

Parasite densities in anti-VAR2CSA-naïve primigravidae decrease following a drastic malaria decline, independently of acquired immunity. This is in contrast with the reductions of immunity among secundigravidae which is accompanied by increasing parasite densities. The effect observed in first-time pregnant women, who have not yet developed pregnancy-specific immunity, might be explained by factors other than acquired immunity that favor low densities in low transmission areas. These observations challenge the dogma that all malaria infections progress to symptomatic infections as immunity wanes with a declining transmission. Both acquired immune and non-immune factors contribute to the substantial heterogeneity in the detectability of *P. falciparum* infections in pregnancy, which is dependent on gravidity and changes in transmission intensity, affecting the sensitivity of current diagnostic tools as well as their role in malaria transmission. Finally, this study emphasizes the need to re-evaluate malaria conceptual frameworks, which are currently centered on the role of immunity, to better inform control and elimination strategies.

## Supplementary Information

**Additional file 1:** **Fig. S1.** Map of Manhiça and Magude District in southern Mozambique where the study was conducted. **Fig. S2:** Performance of the qPCRs and quantitative suspension array assays along with the different experiments. **Fig. S3.** Study profile. **Fig. S4.** Genetic complexity of the infections by study area and period. **Fig. S5.** Antibody levels by studied factors.

**Additional file 2. **Supplementary methods (Quantitative suspension array assay).

**Additional file 3. Table S1.** Characteristics of study participants. **Table S2.** Interactions between centers and study variables on parasitological outcomes. **Table S3.** Interactions between gravidity and temporal trends on parasitological outcomes. **Table S4.** *P. falciparum* parasite rates, density and detectability between centers. **Table S5.** Annual changes in parasitological outcomes in pregnant women from Ilha Josina. **Table S6.** Annual changes in antibody levels by site and gravidity group.

**Additional file 4. **Supplementary results (*P. falciparum* parasite densities).

## Data Availability

A deidentified and restricted dataset, with accompanying data dictionary, can be provided by approved request after completion of a data use agreement, by emailing alfredo.mayor@isglobal.org with the subject line Attention: MiPMon Data.

## Abbreviations

ACS5: Acyl CoA Synthetase 5
ANC: Antenatal care
aOR: Adjusted odds ratio
CI: Confidence interval
COI: Complexity of infection
DBL: Duffy binding-like
DBS: Dried blood spot
EBA175: Erythrocyte-binding antigen-175
ETRAMP5: Early transcribed membrane protein 5
GEXP18: Gametocyte exported protein 18
HSP40: Heat shock protein 40
IgG: Immunoglobulin G
MFIs: Mean fluorescence intensities
MSP119: 19-KDa fragment of the merozoite surface protein-1
pDi: Proportion of detectable infections
PfPR_qPCR_: Quantitative PCR-determined *P. falciparum* positivity rate
PfRH: *P. falciparum* Reticulocyte-binding protein homologue
pI: *p* for the interaction
PfPR_RDT_: Rapid diagnostic test-based *P. falciparum* parasite rate
qPCR: Quantitative real-time polymerase chain-reaction
SD: Standard deviations
SNP: Single nucleotide polymorphism
