# Supplementary material for: Gravidity and malaria trends interact to modify P. falciparum densities and detectability in pregnancy: a 3-year prospective multi-site observational study
Source: BMC Med. 2022 Nov 15;20:396. doi: 10.1186/s12916-022-02597-6 (PMC9664815; doi:10.1186/s12916-022-02597-6)

**Gravidity and malaria trends interact to modify *P. falciparum* densities and detectability in pregnancy: a three-year prospective multi-site observational study**

Glória Matambisso, Nanna Brokhattingen, Sónia Maculuve, Pau Cisteró, Henriques Mbeve, Anna Escoda, Judice Miguel, Elena Buetas, Ianthe de Jong, Boaventura Cuna, Cardoso Melembe, Nelo Ndimande, Gemma Porras, Haily Chen, Kevin K.A. Tetteh, Chris Drakeley, Benoit Gamain, Chetan Chitnis, Virander Chauhan, Llorenç Quintó, Beatriz Galatas, Eusébio Macete, Alfredo Mayor

**Additional File 1: Supplementary figures**

**Figure S1.** Map of Manhiça and Magude District in southern Mozambique where the study was conducted.

**Figure S2.** Performance of the qPCRs and quantitative suspension array assays along with the different experiments.

**Figure S3.**  Study profile.

**Figure S4.**  Genetic complexity of the infections by study area and period.

**Figure S5.** Antibody levels by studied factors.

**Figure S1.** Map of Manhiça and Magude District in Southern Mozambique where the study was conducted.

Green dots represent all the women participating in the study with geographic data information (n=8745).


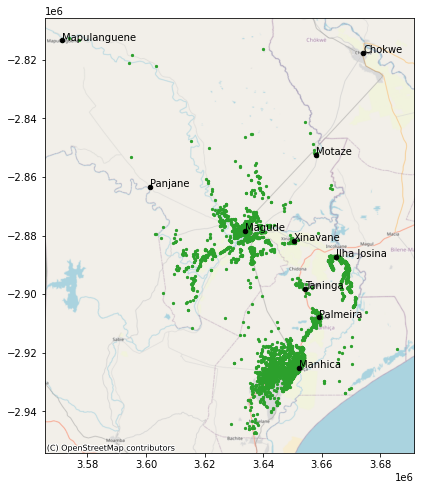


**Figure S2.** Performance of the qPCRs and quantitative suspension array assays along the different experiments.

**A** indicates the parasite density of the third point of the standard curve for each processing plate, **B** the qPCR efficiencies in each plate and **C** the MFI values of the DBS positive control made out of fresh blood and hyperimmune pooled plasma from the standard curve at a concentration of 1:100.


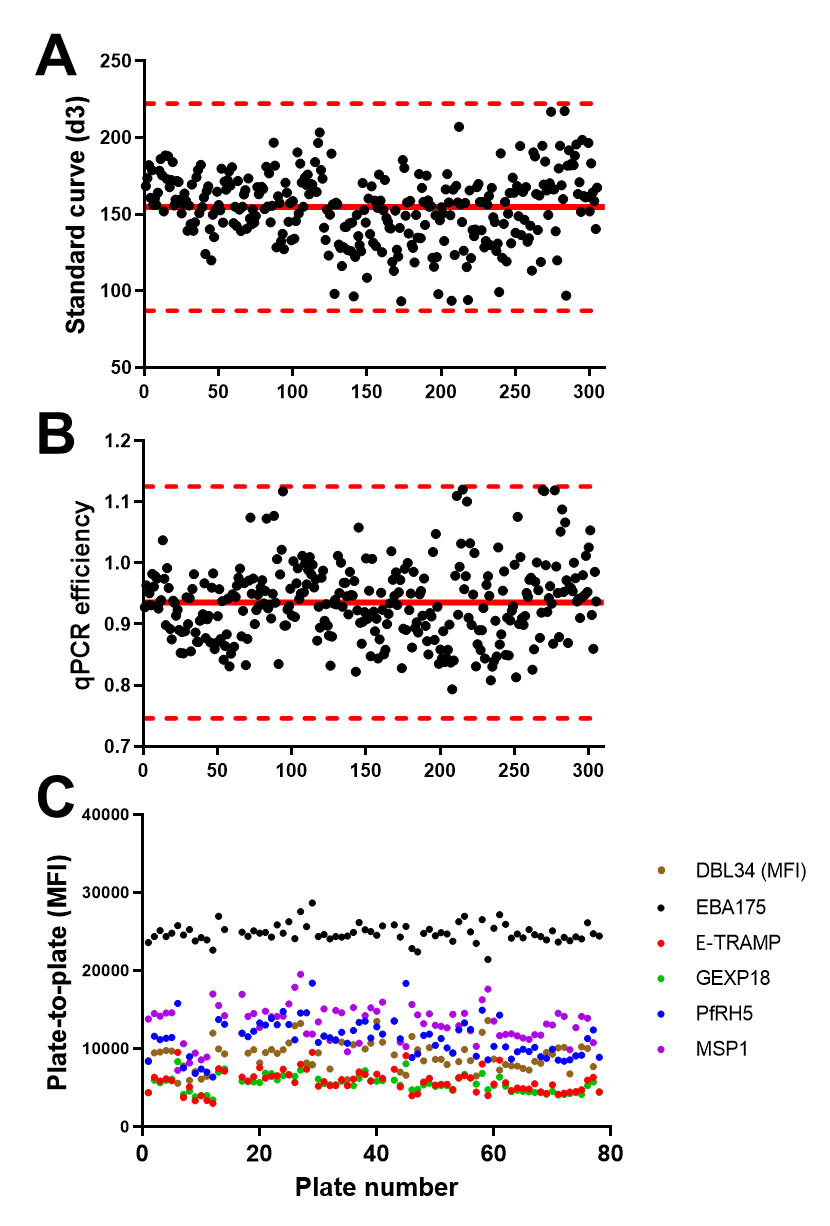


**Figure S3.**  Study profile.


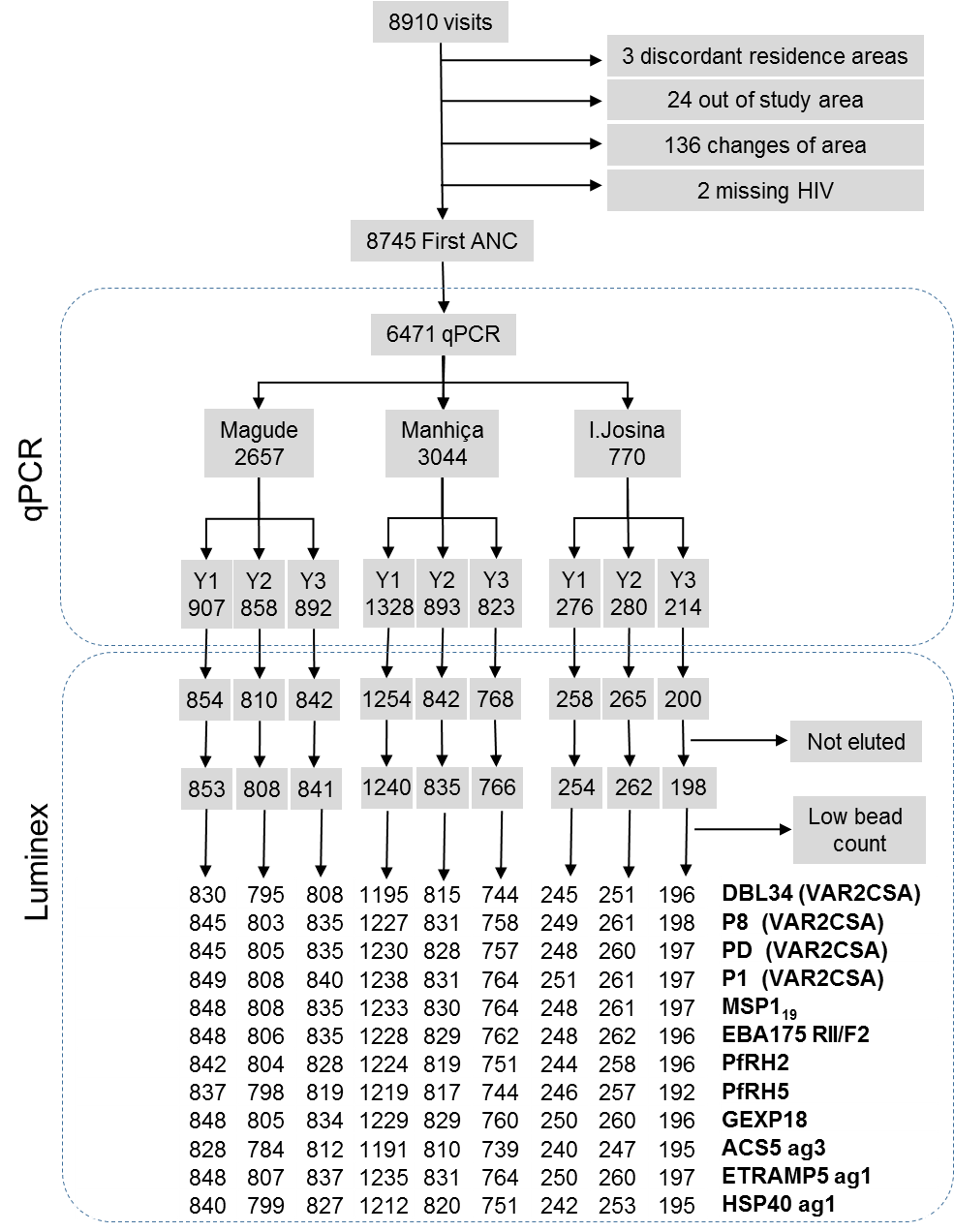


**Figure S4.** Genetic complexity of the infections by study area and period.

Genetic complexity of Infection (COI) was calculated for primigravida (**A**) and multigravidae (**B**) using the programs COIL and represented as violin plots which include a marker for the median (red circle), a black box indicating the interquartile range and spikes extending to the upper- and lower-adjacent values; the shape represents kernel density estimates. Genotyping was successfully achieved in 182 (38%) of the 482 *P. falciparum* infections, with similar rates in the 3 settings (32% [37/115] in Magude, 38% [65/169] in Manhiça and 40% [79/198] in Ilha Josina, p=0.361). P values obtained from the multivariate regression models adjusted by gravidity, season, HIV status, residence in village or rural area and place where molecular analysis was conducted.

**
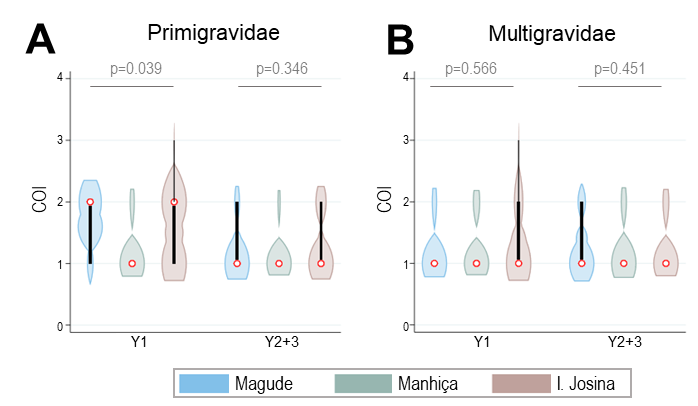
**

**Figure S5.** Antibody levels by studied factors: study clinic (**A**) and period (**B**), gravidity (**C**), *P. falciparum* qPCR status (**D**), HIV status (**E**), residence (**F**), season (**G**) and trimester of gestation at first ANC visit (**H**).

p values obtained from mixed multivariate models of log-transformed MFI values adjusted by all the variables.


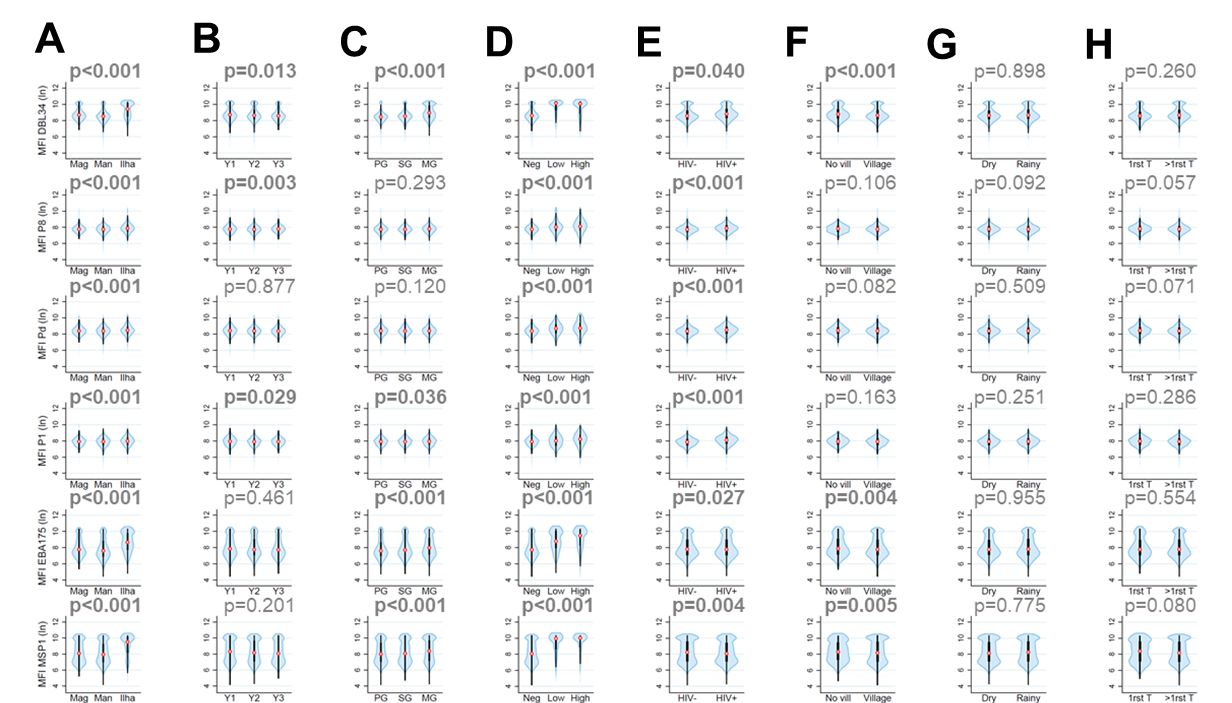


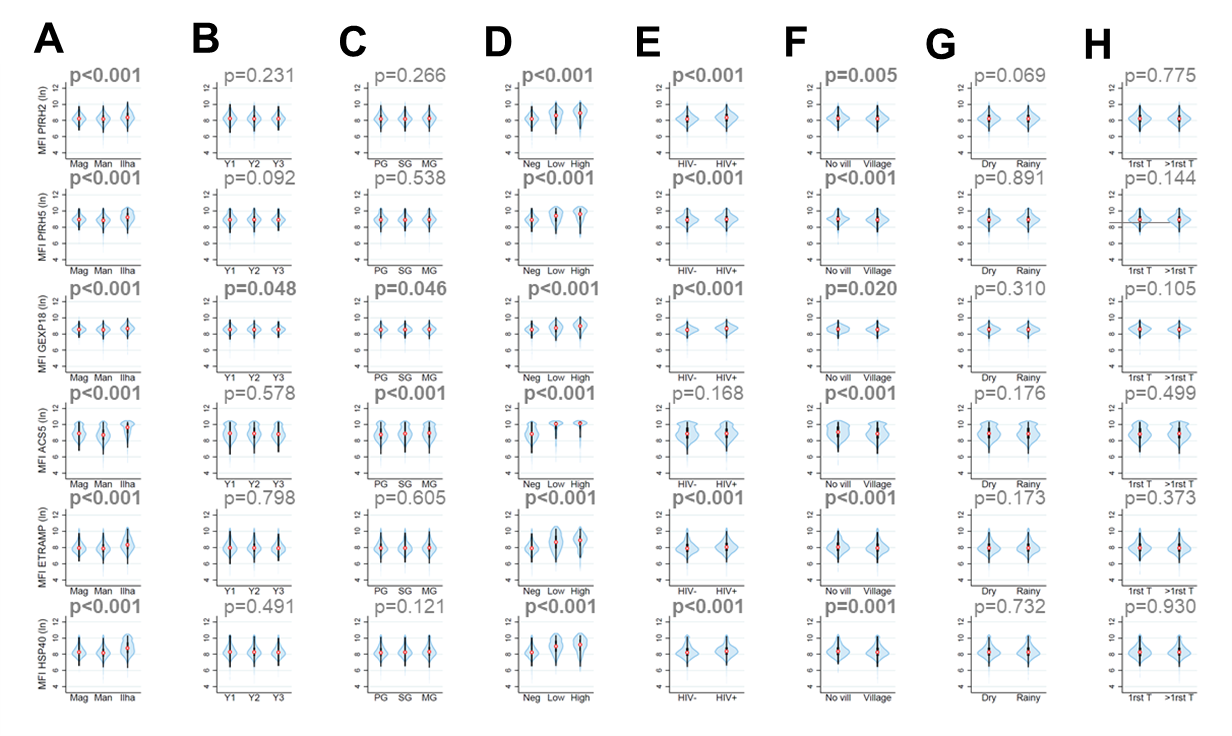

Supplement: Supplementary file 1 — Additional file 1: Fig. S1. Map of Manhiça and Magude District in southern Mozambique where the study was conducted. Fig. S2: Performance of the qPCRs and quantitative suspension array assays along with the different experiments. Fig. S3. Study profile. Fig. S4. Genetic complexity of the infections by study area and period. Fig. S5. Antibody levels by studied factors. [file 12916_2022_2597_MOESM1_ESM.docx]
