# Supplementary material for: Gravidity and malaria trends interact to modify P. falciparum densities and detectability in pregnancy: a 3-year prospective multi-site observational study
Source: BMC Med. 2022 Nov 15;20:396. doi: 10.1186/s12916-022-02597-6 (PMC9664815; doi:10.1186/s12916-022-02597-6)
Supplement: Supplementary file 2 — Additional file 2. Supplementary methods (Quantitative suspension array assay). [file 12916_2022_2597_MOESM2_ESM.docx]

**Gravidity and malaria trends interact to modify *P. falciparum* densities and detectability in pregnancy: a three-year prospective multi-site observational study**

Glória Matambisso, Nanna Brokhattingen, Sónia Maculuve, Pau Cisteró, Henriques Mbeve, Anna Escoda, Judice Miguel, Elena Buetas, Ianthe de Jong, Boaventura Cuna, Cardoso Melembe, Nelo Ndimande, Gemma Porras, Haily Chen, Kevin K.A. Tetteh, Chris Drakeley, Benoit Gamain, Chetan Chitnis, Virander Chauhan, Llorenç Quintó, Beatriz Galatas, Eusébio Macete, Alfredo Mayor

**Additional File 2: Supplementary methods**

**Quantitative polymerase chain reaction**

DNA was extracted from a half-cut (6 punches of 3mm of diameter) of the DBS using the Chelex method [26]. Real-time quantitative PCR (qPCR) targeting *P. falciparum* 18S rRNA on an ABI PRISM 7500 HT Real-Time System (Applied Biosystems) was used (limit of detection: 1-2 parasites/μL). Parasitemia was quantified by extrapolation of cycle thresholds (Ct) from a standard curve of *P. falciparum* ring infected erythrocytes. Samples with no detected Ct were considered negative. The standard curve and negative control with no template DNA were run in all reactions [16]. The performance of the qPCRs was monitored in each experiment by calculating the parasite density of the third point of the standard curve (180 parasite/μL) and the qPCR efficiency. Plates were repeated if these indicators were out of the range obtained by the average +/- 3 standard deviations (**Sup. Fig. 2**).

***P. falciparum* genotyping**

DNA from qPCR-positive DBS was extracted using high-throughput robotic equipment (Qiagen QIAsymphony) at the MalariaGEN Laboratory at the Wellcome Sanger Institute (WSI), Hinxton, UK. After parasite selective whole genome amplification [27], specific genome segments amplified by PCR were sequenced using the Illumina platform. The sequence reads were mapped against the *P. falciparum* 3D7 v3 reference genome and genetic barcodes were constructed by concatenating the alleles at 101 single nucleotide polymorphisms (SNPs) distributed across all nuclear chromosomes [17]. Sample barcodes were used to estimate the number of distinct genotypes within the infection (complexity of Infection [COI]) using the program COIL [18].

**Quantitative suspension array assay**

*Coupling of antigens:* Antigens (see **Table**) were covalently coupled to beads following a modification of the Luminex® Corporation protocol [19]. Briefly, 2 ml of beads (25x10^6^) were transferred into two 1.5 mL Eppendorf tubes and resuspended by sonication and vortexing. The supernatant was removed after precipitation of the beads by magnetic separation for 60 seconds. Beads were washed twice with 1.25 ml of distilled water and pellets were resuspended in 400 μl of activation buffer (0.1 M NaH2PO4, pH 6.2). Sulfo-NHS (N-hydroxysulfosuccinimide) and EDC (1-Ethyl- 3-[3-dimethylaminopropyl] carbodiimide hydrochloride; Pierce, Thermo Fisher Scientific Inc., Rockford, IL) dissolved in activation buffer were simultaneously added to reaction tubes at 5 mg/mL, and reaction tubes were incubated for 20 min at room temperature (RT) with gentle agitation and protected from light. Activated beads were washed twice with 1.25 μl of coupling buffer (MES 50 mM, 2-[Nmorpholino] ethanesulfonic acid monohydrate pH 5, Sigma-Aldrich). Recombinant antigens were coupled to the beads at 30 μg/ml, except peptides and GEXP18 which were coupled at 58 μg/ml and 1.7 mg/ml, respectively. Beads and antigens were vortexed, sonicated and then incubated overnight at 4°C in the dark, with shaking. Coupled beads were blocked with 1.25 ml 1% BSA in PBS for 30 minutes on a shaker at RT protected from light. Subsequently, beads were washed twice with a 1.25 ml assay buffer (1% BSA, 0.05% sodium azide in PBS filtrated) and resuspended in 1.25 ml of the same buffer for a final concentration of 10.000 beads/μl. Beads were quantified on a Guava PCA desktop cytometer (Guava, Hayward, CA), and stored in multiplex at 4°C in the dark.

**Table.** Antigens used to couple the magnetic microspheres and the respective distributor.

| **Antigen** | **Rationale** | **Antigen** | **Producer** | **Ref** |
| --- | --- | --- | --- | --- |
| EBA175 region II F2 | Merozoite antigens | Recombinant | ICGEB, Chetan Chitnis | [22] |
| MSP1_19_ 3D7 |  | Recombinant | ICGEB, Virander Chauhan | [21] |
| RH2 (2030) |  | Recombinant | ICGEB, Deepak Gaur | [23] |
| RH5 |  | Recombinant | ICGEB, Deepak Gaur | [24] |
|  |  |  |  |  |
| VAR2CSA DBL3-4 | Pregnancy-specific (VAR2CSA) | Recombinant | Benoit Gamain | [20] |
| P1 (NTS) |  | Peptide | GL BioChem | [19] |
| P8 (ID1) |  | Peptide | GL BioChem | [19] |
| Pd (ID1) |  | Peptide | GL BioChem | [19] |
|  |  |  |  |  |
| GEXP18 | Recent exposure | Recombinant | LSHTM, Chris Drakeley, Kevin Tetteh | [25] |
| ETRAMP5 ag1 |  | Recombinant | LSHTM, Chris Drakeley, Kevin Tetteh | [25] |
| HSP40 ag1 |  | Recombinant | LSHTM, Chris Drakeley, Kevin Tetteh | [25] |
| ACS5 ag3 |  | Recombinant | LSHTM, Chris Drakeley, Kevin Tetteh | [25] |

*DBS elution:* DBS samples consist of a drop of blood dried onto a filter paper, from which small discs of a sample can later be cut and eluted. 3 mm discs containing approximately 2 μl of blood were obtained using an automated DBS punching machine (DBS Card Punch Machine, Analytical Sales & Services). 1 punched disc per sample was eluted in 50 μl Luminex Buffer in 96-well plates and placed onto a shaker at 4ºC overnight. Quality of elution was evaluated visually, and only samples with white filter paper and red elution were considered well eluted. Eluted samples were diluted 1:4 in Luminex Buffer for a final blood dilution of 1:100, and stored at 4ºC for less than a week until the immunoassay was performed.

*Bead-based immunoassay:* Six controls were included in each plate. 1) To monitor the DBS punching and elution process, mock DBS made from plasma mixed with pooled serum from 45 malaria-immune Mozambican pregnant women. 2) A standard curve was prepared from serum pooled from 35 malaria-immune Mozambican pregnant women in a 3-fold 14-point serial dilution starting at 1:100 with Luminex Buffer (Phosphate Buffered Saline [PBS], 1% BSA, 0.05% azide, pH 7.4). 3) Three dilutions of the standard curve (1:500, 1:5,000 and 1:50,000) were furthermore included to monitor variations between plates (referred to as plate-to-plate controls). 4) As a reference control, National Institute for Biological Standards and Controls (NIBSC) serum against *P. falciparum* (First WHO Reference Reagent for *Pf* anti-malaria human serum, NIBSC 10/198) was included at 1:100 dilution in dH_2_O. Furthermore, 5) negative control samples collected from malaria-naïve individuals and 6) blank wells were included in all plates. To measure the unspecific binding of antibodies in the sample to BSA used to block the beads, beads coupled to BSA were included in the bead multiplex.

50 µl of samples and controls were transferred to Luminex plates. Stock coupled beads were vortexed and diluted to 1,000 beads/well in Luminex Buffer, followed by 1 min bath sonication. 50 µl bead solution was loaded into each well and gently mixed by slow vortexing. Plates were incubated overnight at 4 ºC and 600 rpm on a shaker. The next day, plates were brought back to RT by agitation at 600 rpm for 1 h, before, plates were washed thrice in wash buffer (PBS + 0.05% Tween-20 v/v (Sigma)). For the first wash, 100 µl wash buffer/well was added and the plates were placed onto a magnet for 2 minutes. For the second and third wash, 200 µl/well was added and the plates were placed onto the magnet for 1 minute. Wash buffer was discarded by flicking the plates. Detection antibody (Fc-specific anti-human IgG, Sigma-Aldrich) diluted to 1 µg/ml in Luminex Buffer was added at 100 µl/well, vortexed gently, and plates were incubated for 2 h at 600 rpm at RT. Plates were washed again using the procedure described above. Then streptavidin-phycoerythrin (Sigma-Aldrich) diluted 1:1000 in Luminex Buffer was added at 100 µl/well, and plates were incubated for 45 minutes at 600 rpm at RT. Plates were washed again, 100 μl/well of Luminex Buffer was added, and plates were kept at 4 ºC. The following day, plates were read on Luminex® 100/200^TM^ machines. Acquisition of Luminex raw data was obtained using XPonent software. Protocols were created with the following parameters: MagPlex beads, maximum time per well: 60 seconds, High Photomultiplier Tube acquisition and Doublet Discriminator Gating from 5000 to 25000 nm. Measurements were discarded if fewer than 20 counts per recombinant antigen and 10 per peptide were obtained. Median fluorescence intensities (MFI) were considered valid if two negative and the four positive controls were in the expected range (**Sup. Fig. 2**), otherwise, the plate was repeated.

*Performance of the Luminex assay:* After the elution process of the 4,475 collected DBS, 21 samples were discarded by visual inspection of the elution. The remaining 4,454 samples were analyzed with the Luminex assay, generating a total of 80,172 measurements. 358 of the samples were from women with a qPCR-confirmed *P. falciparum* infection. 1,643 measurements were discarded because of low bead counts. The percentage of covariance between days was calculated from the plate-to-plate controls and considered acceptable if below 30%. It was 13.3% for DBL34, 17.7% for GEXP18, 16.5% for PfRH5, 13.8% for MSP1_19_, 3.1% for EBA175 RII/F2, and 17.3% for ETRAMP5 ag1.
