# Supplementary material for: Gravidity and malaria trends interact to modify P. falciparum densities and detectability in pregnancy: a 3-year prospective multi-site observational study
Source: BMC Med. 2022 Nov 15;20:396. doi: 10.1186/s12916-022-02597-6 (PMC9664815; doi:10.1186/s12916-022-02597-6)
Supplement: Supplementary file 3 — Additional file 3. Table S1. Characteristics of study participants. Table S2. Interactions between centers and study variables on parasitological outcomes. Table S3. Interactions between gravidity and temporal trends on parasitological outcomes. Table S4. P. falciparum parasite rates, density and detectability between centers. Table S5. Annual changes in parasitological outcomes in pregnant women from Ilha Josina. Table S6. Annual changes in antibody levels by site and gravidity group. [file 12916_2022_2597_MOESM3_ESM.docx]

**Gravidity and malaria trends interact to modify *P. falciparum* densities and detectability in pregnancy: a three-year prospective multi-site observational study**

Glória Matambisso, Nanna Brokhattingen, Sónia Maculuve, Pau Cisteró, Henriques Mbeve, Anna Escoda, Judice Miguel, Elena Buetas, Ianthe de Jong, Boaventura Cuna, Cardoso Melembe, Nelo Ndimande, Gemma Porras, Haily Chen, Kevin K.A. Tetteh, Chris Drakeley, Benoit Gamain, Chetan Chitnis, Virander Chauhan, Llorenç Quintó, Beatriz Galatas, Eusébio Macete, Alfredo Mayor

**Additional File 3: Supplementary table**

**Table S1.** Characteristics of women who were included for qPCR analysis and all women recruited during the three years of recruitment, and baseline characteristics of the women with qPCR result in accordance to the study area.

**Table S2.** Interactions between centers and study variables on parasitological outcomes.

**Table S3.** Interactions between gravidity and temporal trends on parasitological outcomes by a health clinic.

**Table S4.** *P. falciparum* parasite rates, density and detectability between centers by gravidity and study period.

**Table S5.** Annual changes in parasitological outcomes in pregnant women from Ilha Josina by gravidity group.

**Table S6.** Annual changes in antibody levels by site and gravidity group.

**Table S1.** Characteristics of women who were included for qPCR analysis and all women recruited during the three years of recruitment, and baseline characteristics of the women with qPCR result in accordance to the study area.

|  |  | **Recruited vs qPCR done** | | | | |  | **qPCR result available** | | | | | | | |
| --- | --- | --- | --- | --- | --- | --- | --- | --- | --- | --- | --- | --- | --- | --- | --- |
|  |  | **Recruited** | |  | **qPCR** | |  | **Magude** | |  | **Manhiça** | |  | **Ilha Josina** | |
|  |  | **n=8745** | |  | **n=6471** | |  | **n=2657** | |  | **n=3044** | |  | **n=770** | |
|  |  | **n** | **%** |  | **n** | **%** |  | **n** | **%** |  | **n** | **%** |  | **n** | **%** |
| **Study period** | |  |  |  |  |  |  |  |  |  |  |  |  |  |  |
|  | **Nov 16-Oct 17** | 2783 | 32 |  | 2511 | 39 |  | 907 | 34 |  | 1328 | 44 |  | 276 | 36 |
|  | **Nov 17-Oct 18** | 3021 | 35 |  | 2031 | 31 |  | 858 | 32 |  | 893 | 29 |  | 280 | 36 |
|  | **Nov 18-Oct 19** | 2941 | 43 |  | 1929 | 30 |  | 892 | 34 |  | 823 | 27 |  | 214 | 28 |
| **Clinic** | |  |  |  |  |  |  |  |  |  |  |  |  |  |  |
|  | **Magude** | 3305 | 38 |  | 2657 | 41 |  | 2657 | 100 |  | 0 | 0 |  | 0 | 0 |
|  | **Manhiça** | 4643 | 53 |  | 3044 | 47 |  | 0 | 0 |  | 3044 | 100 |  | 0 | 0 |
|  | **Ilha Josina** | 797 | 9 |  | 770 | 12 |  | 0 | 0 |  | 0 | 0 |  | 770 | 100 |
| **Village** | |  |  |  |  |  |  |  |  |  |  |  |  |  |  |
|  | **Yes** | 8142 | 93 |  | 6018 | 93 |  | 2459 | 93 |  | 2789 | 92 |  | 770 | 100 |
|  | **No** | 603 | 7 |  | 453 | 7 |  | 198 | 7 |  | 255 | 8 |  | 0 | 0 |
| **Season** | |  |  |  |  |  |  |  |  |  |  |  |  |  |  |
|  | **Rainy** | 4500 | 51 |  | 3390 | 52 |  | 1319 | 50 |  | 1654 | 54 |  | 417 | 54 |
|  | **Dry** | 4245 | 49 |  | 3081 | 48 |  | 1338 | 50 |  | 1390 | 46 |  | 353 | 46 |
| **Age (years)** | |  |  |  |  |  |  |  |  |  |  |  |  |  |  |
|  | **<18** | 927 | 11 |  | 670 | 10 |  | 279 | 11 |  | 267 | 9 |  | 124 | 16 |
|  | **>=18** | 7818 | 89 |  | 5801 | 90 |  | 2378 | 89 |  | 2777 | 91 |  | 646 | 84 |
| **Gravidity** | |  |  |  |  |  |  |  |  |  |  |  |  |  |  |
|  | **Primigravidae** | 2458 | 28 |  | 1754 | 27 |  | 727 | 27 |  | 824 | 27 |  | 203 | 26 |
|  | **Multigravidae** | 6287 | 72 |  | 4717 | 73 |  | 1930 | 73 |  | 2220 | 73 |  | 567 | 74 |
| **Trimester** | |  |  |  |  |  |  |  |  |  |  |  |  |  |  |
|  | **1st** | 732 | 8 |  | 518 | 8 |  | 144 | 5 |  | 316 | 10 |  | 58 | 8 |
|  | **>1st** | 8013 | 92 |  | 5953 | 92 |  | 2513 | 95 |  | 2728 | 90 |  | 712 | 92 |
| **HIV** | |  |  |  |  |  |  |  |  |  |  |  |  |  |  |
|  | **Positive** | 2557 | 29 |  | 1872 | 29 |  | 714 | 27 |  | 962 | 32 |  | 196 | 25 |
|  | **Negative** | 6188 | 71 |  | 4599 | 71 |  | 1943 | 73 |  | 2082 | 68 |  | 574 | 75 |

**Table S2.** Interactions between centers and study variables on parasitological outcomes.

Multivariate logistic models for *Pf* positivity rate and detectability and linear models for log-transformed parasite densities, including as variables study period (2016 to 2019), gravidity (primi and multigravidae), HIV status, season and trimester at first antenatal care visit, residence in a village or rural area and place where molecular analysis was conducted (Mozambique or Spain). The modification of the associations was assessed by including interaction terms into the regression models and combining the coefficients plus the interaction and the standard error by the delta method. ARC, which expresses the annual relative change in the parasitological outcome per year was computed as the exponential of the linear combination of the main effects plus their interactions; 95% confidence intervals were calculated based on the delta method standard errors. The statistical significance of the interaction term (p for the interaction [pI]) was assessed using a Wald test.

|  |  | ***P. falciparum* positivity (by qPCR)** | | | |  | ***Pf* density** | | | |  | **Proportion Detectable infections** | | | |
| --- | --- | --- | --- | --- | --- | --- | --- | --- | --- | --- | --- | --- | --- | --- | --- |
|  |  | **aRC** | **95%CI** | **p** | **pI** |  | **aRC** | **95%CI** | **p** | **pI** |  | **aRC** | **95%CI** | **p** | **pI** |
| **Trend** | |  |  |  |  |  |  |  |  |  |  |  |  |  |  |
|  | Magude | 0.56 | 0.44; 0.71 | **<0.001** | 0.956 |  | 1.99 | 0.93; 4.11 | **0.060** | **0.098** |  | 1.57 | 0.95; 2.62 | **0.081** | 0.220 |
|  | Manhiça | 0.55 | 0.45; 0.67 | **<0.001** |  |  | 0.74 | 0.42; 1.39 | 0.315 |  |  | 0.94 | 0.63; 1.44 | 0.784 |  |
|  | Ilha Josina | 0.58 | 0.47; 0.72 | **<0.001** |  |  | 1.20 | 0.67; 2.17 | 0.548 |  |  | 0.96 | 0.64; 1.46 | 0.852 |  |
| **Gravidity** | |  |  |  |  |  |  |  |  |  |  |  |  |  |  |
|  | Magude | 0.57 | 0.38; 0.84 | **0.005** | 0.622 |  | 0.16 | 0.05; 0.51 | **0.002** | **0.009** |  | 0.31 | 0.39; 0.69 | **0.004** | **0.010** |
|  | Manhiça | 0.71 | 0.50; 0.98 | **0.045** |  |  | 0.52 | 0.19; 1.42 | 0.195 |  |  | 0.47 | 0.25; 0.94 | **0.029** |  |
|  | Ilha Josina | 0.57 | 0.40; 0.83 | **0.003** |  |  | 0.06 | 0.03; 0.16 | **<0.001** |  |  | 0.11 | 0.06; 0.22 | **<0.001** |  |
| **HIV** | |  |  |  |  |  |  |  |  |  |  |  |  |  |  |
|  | Magude | 0.77 | 0.49; 1.12 | 0.273 | 0.880 |  | 1.96 | 0.49; 7.84 | 0.340 | 0.211 |  | 1.98 | 0.77; 5.06 | 0.156 | 0.718 |
|  | Manhiça | 0.87 | 0.60; 1.22 | 0.456 |  |  | 6.74 | 2.52; 20.99 | **<0.001** |  |  | 2.38 | 0.19; 5.07 | **0.019** |  |
|  | Ilha Josina | 0.78 | 0.52; 1.16 | 0.213 |  |  | 2.06 | 0.71; 5.92 | 0.180 |  |  | 1.56 | 0.76; 3.20 | 0.226 |  |
| **Rainy** | |  |  |  |  |  |  |  |  |  |  |  |  |  |  |
|  | Magude | 1.19 | 0.80; 1.76 | 0.397 | 0.806 |  | 0.69 | 0.21; 2.28 | 0.536 | 0.406 |  | 0.97 | 0.43; 2.21 | 0.933 | 0.306 |
|  | Manhiça | 1.01 | 0.72; 1.39 | 0.952 |  |  | 1.41 | 0.54; 3.98 | 0.501 |  |  | 1.43 | 0.74; 2.95 | 0.314 |  |
|  | Ilha Josina | 1.14 | 0.80; 1.63 | 0.476 |  |  | 1.85 | 0.71; 4.74 | 0.201 |  |  | 2.14 | 1.09; 4.15 | **0.025** |  |
| **Trimester** | |  |  |  |  |  |  |  |  |  |  |  |  |  |  |
|  | Magude | 0.70 | 0.32; 1.55 | 0.381 | 0.353 |  | 0.09 | 0.01; 0.94 | **0.043** | 0.138 |  | 0.46 | 0.09; 2.26 | 0.898 | 0.494 |
|  | Manhiça | 1.42 | 0.78; 2.61 | 0.259 |  |  | 0.46 | 0.07; 2.72 | 0.399 |  |  | 0.53 | 1.42; 1.95 | 0.316 |  |
|  | Ilha Josina | 0.93 | 0.50; 1.72 | 0.810 |  |  | 1.52 | 0.31; 7.38 | 0.604 |  |  | 1.24 | 0.41; 3.75 | 0.326 |  |

**Table S3.** Interactions between gravidity and temporal trends on parasitological outcomes by a health clinic.

The modification of the associations by gravidity was assessed by including interaction terms into the regression models and combining the coefficients plus the interaction and the standard error by the delta method.

P values were obtained from multivariate regression models predicting parasitological outcome in each of the three sites based on visit date, gravidity and their two-way interactions, adjusted by season, HIV status, residence in a village or rural area and place where molecular analysis was conducted. aRC, which expresses the annual relative change in the parasitological outcome per year was computed as the exponential of the linear combination of the main effects plus their interactions; 95% confidence intervals were calculated based on the delta method standard errors. The statistical significance of the interaction term (p for the interaction [pI]) was assessed using a Wald test.

|  |  |  |  | **aRC** | **95%CI** | **p** | **pI** |
| --- | --- | --- | --- | --- | --- | --- | --- |
| **Magude** | | | |  |  |  |  |
|  | ***P. falciparum* positivity (by qPCR)** | | | | |  |  |
|  |  |  | Primigravidae | 0.58 | 0.35; 0.80 | **0.006** | 0.908 |
|  |  |  | Multigravidae | 0.56 | 0.38; 0.73 | **<0.001** |  |
|  | **Parasite density** | | |  |  |  |  |
|  |  |  | Primigravidae | 1.71 | 0.39; 3.81 | 0.391 | 0.583 |
|  |  |  | Multigravidae | 2.66 | 0.44; 5.76 | 0.100 |  |
|  | **Proportion Detectable infections** | | | |  |  |  |
|  |  |  | Primigravidae | 1.39 | 0.33; 2.46 | 0.394 | 0.692 |
|  |  |  | Multigravidae | 1.70 | 0.46; 2.94 | 0.154 |  |
|  |  |  |  |  |  |  |  |
| **Manhiça** | | | |  |  |  |  |
|  | ***P. falciparum* positivity (by qPCR)** | | | | |  |  |
|  |  |  | Primigravidae | 0.64 | 0.43; 0.85 | **0.008** | 0.209 |
|  |  |  | Multigravidae | 0.49 | 0.36; 0.62 | **<0.001** |  |
|  | **Parasite density** | | |  |  |  |  |
|  |  |  | Primigravidae | 0.76 | 0.06; 1.45 | 0.554 | 0.894 |
|  |  |  | Multigravidae | 0.82 | 0.15; 1.49 | 0.636 |  |
|  | **Proportion Detectable infections** | | | |  |  |  |
|  |  |  | Primigravidae | 1.00 | 0.39; 1.61 | 0.994 | 0.856 |
|  |  |  | Multigravidae | 0.93 | 0.42; 1.45 | 0.806 |  |
|  |  |  |  |  |  |  |  |
| **Ilha Josina** | | | |  |  |  |  |
|  | ***P. falciparum* positivity (by qPCR)** | | | | |  |  |
|  |  |  | Primigravidae | 0.55 | 0.33; 0.77 | **0.003** | 0.703 |
|  |  |  | Multigravidae | 0.60 | 0.44; 0.76 | **<0.001** |  |
|  | **Parasite density** | | |  |  |  |  |
|  |  |  | Primigravidae | 0.66 | 0.06; 1.26 | 0.373 | **0.079** |
|  |  |  | Multigravidae | 1.73 | 0.61; 2.86 | **0.095** |  |
|  | **Proportion Detectable infections** | | | |  |  |  |
|  |  |  | Primigravidae | 0.41 | 0.08; 0.73 | **0.029** | **0.007** |
|  |  |  | Multigravidae | 1.47 | 0.68; 2.25 | 0.161 |  |

**Table S4.** *P. falciparum* parasite rates, density and detectability between centers by gravidity and study period.

Detectable infections are considered those with parasite densities above 100 parasites/µL (n) and expressed relative to the total of infections detected by qPCR (N). p values were obtained from multivariate logistic models for *P. falciparum* positivity rate and proportion of detectable infections, and linear models for log-transformed parasite densities, adjusted by gravidity, season, HIV status, residence in a village or rural area and place where molecular analysis was conducted (Mozambique or Spain). The model included an interaction term to assess the modifying effects of gravidity on the relationship between parasitological outcomes and center of recruitment, with the testing for significance using a Wald test (pI).

|  |  |  | ***P. falciparum* positivity (by qPCR)** | | | | |  | ***P. falciparum* densities** | | | | |  | **Proportion Detectable infections** | | | | |
| --- | --- | --- | --- | --- | --- | --- | --- | --- | --- | --- | --- | --- | --- | --- | --- | --- | --- | --- | --- |
|  |  |  | **N** | **n** | **%** | **p** | **pI** |  | **N** | **GM** | **SD** | **p** | **pI** |  | **N** | **n** | **%** | **p** | **pI** |
| **Nov 2016-October 2017** | | | |  |  |  |  |  |  |  |  |  |  |  |  |  |  |  |  |
|  | **Primigravidae** | |  |  |  |  |  |  |  |  |  |  |  |  |  |  |  |  |  |
|  |  | **Magude** | 182 | 18 | 10 | **<0.001** | 0.600 |  | 18 | 57.3 | 195.5 | 0.116 | **0.022** |  | 18 | 9 | 50 | **0.048** | **0.008** |
|  |  | **Manhiça** | 317 | 33 | 10 |  |  |  | 33 | 72.4 | 210.9 |  |  |  | 33 | 19 | 58 |  |  |
|  |  | **Ilha Josina** | 64 | 29 | 45 |  |  |  | 29 | 253.0 | 729.0 |  |  |  | 29 | 24 | 83 |  |  |
|  | **Multigravidae** | |  |  |  |  |  |  |  |  |  |  |  |  |  |  |  |  |  |
|  |  | **Magude** | 725 | 40 | 6 | **<0.001** |  |  | 40 | 9.1 | 31.1 | **0.013** |  |  | 40 | 10 | 25 | **0.049** |  |
|  |  | **Manhiça** | 1011 | 78 | 8 |  |  |  | 78 | 43.9 | 146.2 |  |  |  | 78 | 34 | 44 |  |  |
|  |  | **Ilha Josina** | 212 | 70 | 33 |  |  |  | 70 | 10.8 | 29.4 |  |  |  | 70 | 15 | 21 |  |  |
|  |  |  |  |  |  |  |  |  |  |  |  |  |  |  |  |  |  |  |  |
|  |  |  |  |  |  |  |  |  |  |  |  |  |  |  |  |  |  |  |  |
| **Nov 2017-October 2019** | | | | | | | | | | | | | | | | | | | |
|  | **Primigravidae** | |  |  |  |  |  |  |  |  |  |  |  |  |  |  |  |  |  |
|  |  | **Magude** | 545 | 24 | 4 | **<0.001** | 0.947 |  | 24 | 118.9 | 419.1 | 0.277 | 0.324 |  | 24 | 16 | 67 | 0.529 | 0.477 |
|  |  | **Manhiça** | 507 | 24 | 5 |  |  |  | 24 | 48.8 | 164.8 |  |  |  | 24 | 13 | 54 |  |  |
|  |  | **Ilha Josina** | 139 | 39 | 28 |  |  |  | 39 | 136.9 | 359.4 |  |  |  | 39 | 25 | 64 |  |  |
|  | **Multigravidae** | |  |  |  |  |  |  |  |  |  |  |  |  |  |  |  |  |  |
|  |  | **Magude** | 1205 | 33 | 3 | **<0.001** |  |  | 33 | 36.4 | 127.9 | 0.515 |  |  | 33 | 15 | 45 | 0.293 |  |
|  |  | **Manhiça** | 1209 | 34 | 3 |  |  |  | 34 | 29.5 | 95.2 |  |  |  | 34 | 13 | 38 |  |  |
|  |  | **Ilha Josina** | 355 | 61 | 17 |  |  |  | 61 | 19.7 | 59.6 |  |  |  | 61 | 19 | 31 |  |  |
| PfPR, *P. falciparum* parasite rate; GM, geometric mean; SD, Standard deviation; pI, p value for the interaction | | | | | | | | | | | | | | | |  |  |  |  |

**Table S5.** Annual changes in parasitological outcomes in pregnant women from Ilha Josina by gravidity group.

P values were obtained from multivariate regression models predicting parasitological outcome in Ilha Josina based on visit date, gravidity, and their two-way interactions, adjusted by season, HIV status, residence in village or rural area and place where molecular analysis was conducted. The statistical significance of the interaction term (p for the interaction [pI]) was assessed using a Wald test. aRC, which expresses the annual relative change in the parasitological outcome per year was computed as the exponential of the linear combination of the main effects plus their interactions; 95% confidence intervals were calculated based on the delta method standard errors. The statistical significance of the interaction term (p for the interaction [Wald pI]) was assessed using a Wald test.

|  |  | **aRC** | **95%CI** | **p** | **pI** |
| --- | --- | --- | --- | --- | --- |
| **qPCR positivity** | |  |  |  |  |
|  | Primigravidae | 0.54 | 0.37; 0.81 | **0.003** | 0.942 |
|  | Secundigravidae | 0.59 | 0.37; 0.94 | **0.027** |  |
|  | Multigravidae | 0.59 | 0.44; 0.80 | **0.001** |  |
| ***Pf* density** | |  |  |  |  |
|  | Primigravidae | 0.62 | 0.25; 1.55 | 0.305 | **0.043** |
|  | Secundigravidae | 4.63 | 1.28; 16.82 | **0.020** |  |
|  | Multigravidae | 1.13 | 0.55; 2.31 | 0.736 |  |
| **Proportion Detectable infections** | | |  |  |  |
|  | Primigravidae | 0.41 | 0.18; 0.91 | **0.028** | **0.019** |
|  | Secundigravidae | 2.43 | 0.87; 6.76 | 0.089 |  |
|  | Multigravidae | 1.14 | 0.60; 2.16 | 0.691 |  |

aRC, annual relative change

**Table S6.** Annual changes in antibody levels by site and gravidity group.

P values were obtained from multivariate regression models predicting antibody levels in Magude, Manhiça and Ilha Josina based on visit date, gravidity, and their two-way interactions, adjusted by season, HIV status, residence in village or rural area. The statistical significance of the interaction term (p for the interaction [pI]) was assessed using a Wald test. aRC express the annual relative change in the parasitological outcome and its 95% confidence interval which were estimated by the delta method.
