# Supplementary material for: Gravidity and malaria trends interact to modify P. falciparum densities and detectability in pregnancy: a 3-year prospective multi-site observational study
Source: BMC Med. 2022 Nov 15;20:396. doi: 10.1186/s12916-022-02597-6 (PMC9664815; doi:10.1186/s12916-022-02597-6)
Supplement: Supplementary file 4 — Additional file 4. Supplementary results (P. falciparum parasite densities). [file 12916_2022_2597_MOESM4_ESM.docx]

**Gravidity and malaria trends interact to modify *P. falciparum* densities and detectability in pregnancy: a three-year prospective multi-site observational study**

Glória Matambisso, Nanna Brokhattingen, Sónia Maculuve, Pau Cisteró, Henriques Mbeve, Anna Escoda, Judice Miguel, Elena Buetas, Ianthe de Jong, Boaventura Cuna, Cardoso Melembe, Nelo Ndimande, Gemma Porras, Haily Chen, Kevin K.A. Tetteh, Chris Drakeley, Benoit Gamain, Chetan Chitnis, Virander Chauhan, Llorenç Quintó, Beatriz Galatas, Eusébio Macete, Alfredo Mayor

**Additional File 4: Supplementary results**

**Supplementary Results.** Parasite densities

The association between parasite densities and gravidity depended on parasite rates and study period. Parasite densities were lower in multigravidae than primigravidae from Ilha Josina and Magude, but not from Manhiça (pI=0.009; **Sup. Table 2**). At the beginning of the study (November 2016 to October 2017), primigravid women from Ilha Josina carried the highest parasite densities compared to primigravidae from Magude (proportional increase: 3.73, 95%CI [0.60-23.07], p=0.156) and Manhiça (3.87, 95%CI [0.85-17.59], p=0.080). In contrast, densities were the lowest among multigravid women from Manhiça compared to multigravidae from Ilha Josina (0.32, 95%CI [0.11-0.90], p=0.030) and Magude (0.22, 95%CI[0.08-0.73], p=0.013; pI=0.022; **Fig. 1** and **Sup. Table 4**). Thereafter, parasite densities in Ilha Josina tended to decline among primigravid women (average proportional change per year of 0.66, 95%CI [0.06-1.26], p=0.373) and to increase among multigravidae (1.73, 95%CI [0.61-2.86], p=0.095; pI=0.079; **Fig. 2** and **Sup. Table 3**). The exploratory analysis showed that this increase in parasite densities among multigravid women from Ilha Josina was affecting secundigravid women (4.63, 95%CI[1.28-16.82], p=0.020) but not women with 3 or more previous pregnancies (1.13, 95%CI[0.55-2.31], p=0.736 pI=0.043); **Sup. Fig. 5 & Sup. Table 5**). Parasite densities in Manhiça and Magude were maintained stable (0.74, 95%CI [0.30-1.18], p=0.315) or tended to increase, respectively (1.99, 95%CI [0.57-3.40], p=0.060; **Fig. 2** & **Sup. Table 3**, with no evidence of a modification of these trends by gravidity.
